# Novel insights into mitotic chromosome condensation

**DOI:** 10.12688/f1000research.8727.1

**Published:** 2016-07-25

**Authors:** Ewa Piskadlo, Raquel A. Oliveira

**Affiliations:** 1 Instituto Gulbenkian de Ciência, Rua da Quinta Grande 6, Oeiras, 2780-156 , Portugal

**Keywords:** mitotic, mitosis, chromosome, condensation

## Abstract

The fidelity of mitosis is essential for life, and successful completion of this process relies on drastic changes in chromosome organization at the onset of nuclear division. The mechanisms that govern chromosome compaction at every cell division cycle are still far from full comprehension, yet recent studies provide novel insights into this problem, challenging classical views on mitotic chromosome assembly. Here, we briefly introduce various models for chromosome assembly and known factors involved in the condensation process (e.g. condensin complexes and topoisomerase II). We will then focus on a few selected studies that have recently brought novel insights into the mysterious way chromosomes are condensed during nuclear division.

## Introduction: why do chromosomes condense during mitosis?

Mitosis was first described in the 19th century and has captivated generations of scientists ever since. This fascinating process comprises the assembly of interphase chromatin into individual chromosomes and subsequently the equal separation of the genetic material between two daughter cells. Mitosis is undoubtedly an extremely complex operation that needs to be conducted and controlled precisely under the penalty of dismantling genome integrity. One of the key steps in mitosis is chromosome condensation – the compaction of the chromatin into well-defined rod-shaped structures (for other recent reviews, see 1– 3). This process is cytologically very evident, yet both the internal structure of the mitotic chromosomes and the mechanisms by which this transformation is achieved remain quite elusive. To ensure that cell division is feasible within the cell space, vertebrate cells compact their DNA around 2–3 times more than in interphase, as estimated by chromatin volume measurements ^4, 5^ and Förster resonance energy transfer (FRET)-based assays between histones ^6^. Spatial compaction, however, is not the only important outcome of condensation. The structural reorganization during condensation leads to the separation of the identical sister chromatids from each other (known as sister chromatid resolution). Several topological constraints arise throughout interphase (most notably during DNA replication) that result in the entanglement of the two DNA molecules. The resolution of such intertwines (i.e. individualization) is crucial for efficient and faithful chromosome segregation during mitosis. Condensation of chromatin into sturdy chromosomes is also necessary to establish proper physical properties. Chromosomes must be stiff, resilient, and elastic enough to withstand forces coming from pulling microtubules and cytoplasmic drags during mitosis to prevent damage and breaks caused by external tensions.

Despite the utmost importance of chromosome condensation for the fidelity of mitosis, the molecular mechanisms that drive this process remain very unclear. Here we highlight recent findings regarding this process, discussed in the context of different models for mitotic chromosome condensation.

## Models for mitotic chromosome architecture

**Figure 1.**
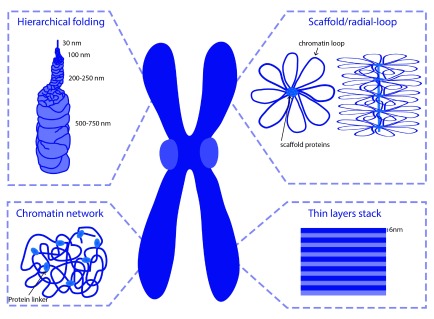
Schematic representation of current models for mitotic chromosome condensation. Adapted from Daban *et al.* ^21^

Over the past few decades, detailed characterization of metaphase chromosomes, using different cytological approaches, has led to the proposal of several models for mitotic chromosome assembly ( Figure 1).

Classical views on chromosome organization postulate that mitotic chromosomes result from chromatin fiber folding. DuPraw suggested that fiber folding occurs randomly, transversely, and longitudinally, with no intermediate levels of compaction ^7^. However, mitotic chromosomes fold into a reproducible structure in every mitosis, at least to some extent. Mitotic chromosomes acquire a reproducible length and display an invariable signature pattern of bands after staining with specific dyes, such as Giemsa. Moreover, specific DNA sequences occupy a reproducible position along the longitudinal and transverse axes of the chromosome ^8^. Although some degree of randomness was observed within chromosomal domains ^9, 10^, chromosome assembly cannot be explained as a purely random process.

Alternatively, it has been suggested that metaphase chromosomes result from helical coiling events (helical-coiling model). The nucleo-histone fiber is proposed to be coiled up into a helix, which is hierarchically wound up into larger helices to achieve the compactness of the mitotic chromosome ( Figure 1) ^11, 12^. This model has been widely accepted, as lower levels of chromatin organization were long postulated to result from hierarchical folding: wrapping of DNA around nucleosomes forms a 11 nm bead-on-a-string structure that coils up into a 30 nm fiber. However, the existence of this 30 nm fiber *in vivo* is yet to be confirmed and has been recently highly debated ^13– 15^.

Using electron microscopy (EM) studies, Paulson and Laemmli ^16^ provided a novel view on chromosome organization. Upon histone removal, chromosomes revealed a scaffold or core that has the shape of intact chromosomes, surrounded by loops of chromatin attached to this central core ^17, 18^. These and subsequent studies led to the consolidation of the scaffold/radial-loop model, which argues that radial DNA loops extend out from a protein element or scaffold positioned along the central axis of the chromatid.

In contrast to the scaffold model, analysis of the biophysical properties of mitotic chromosomes has challenged the idea that the continuity of mitotic chromosomes depends on its proteinaceous core. Taking advantage of the highly elastic behavior displayed by mitotic chromosomes, *in vitro* elasticity measurements revealed that the elastic response of mitotic chromosomes is lost after DNA digestion ^19^. Mild protease treatment, in contrast, does not impair a reversible elastic response despite a progressively reduced force constant ^19, 20^. This led to the proposal of the chromatin-network model, in which chromatin itself is proposed to be the mechanical contiguous component of the mitotic chromosome.

More recent ideas for the internal folding of chromosomes suggest that mitotic chromosomes are arranged into stacks of 6 nm layers ^21^. Those layers would be perpendicular to the chromosome axis and contain around 1 Mb of consequent DNA. Such arrangement of chromosomes has the advantage of explaining properties of G-bands and the geometry of chromosome translocations in a better way than other models.

Despite the differential contributions for chromatin/protein components within chromosome organization, these models might not be mutually exclusive and stacks, coils, and radial loops may co-exist within a less ordered structure.

## Known players of condensation

Despite the several unknowns on the precise molecular details of chromosome assembly, some key components are believed to be crucial for chromosome organization.

### Condensins

**Figure 2.**
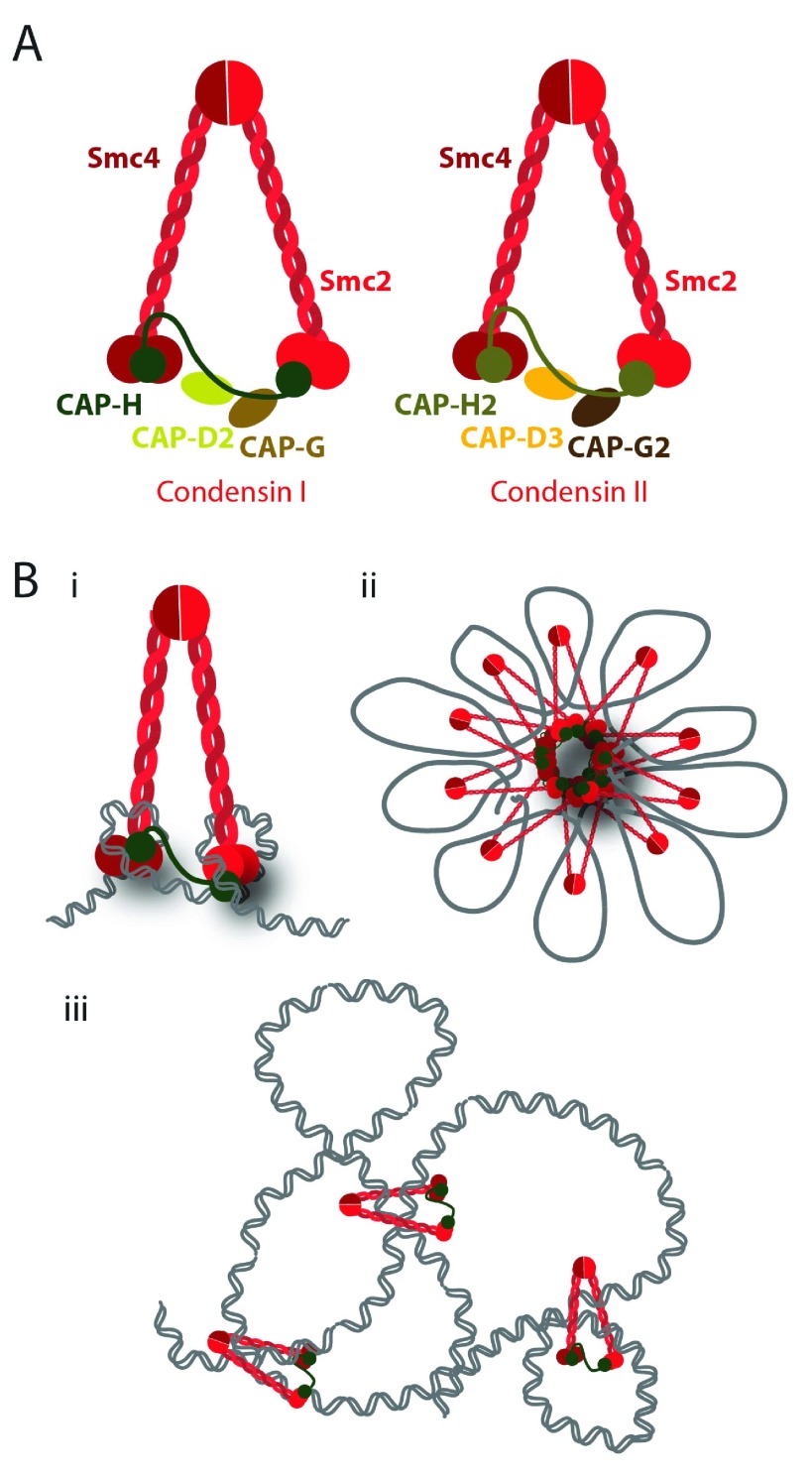
Condensin complexes. **A**) Schematic representation of the structure of condensin complexes. In metazoans, there are two types of condensins, condensin I and condensin II. The SMC2/SMC4 heterodimer is shared by both complexes, while the non-SMC subunits differ: CAP-D2, CAP-G, and CAP-H (Kleisin γ) for condensin I and CAP-D3, CAP-G2, and CAP-H2 (Kleisin β) for condensin II. **B**) Possible models for the condensins’ role in DNA compaction include DNA supercoiling, loop-holder, and topological linker.

Condensins are a conserved group of multi-subunit proteins ( Figure 2a) fulfilling many roles in chromatin organization throughout the cell cycle, but their most prominent function is to ensure efficient chromosome segregation (reviewed in ^22– 24^). They were first isolated from *Xenopus* egg extract, and immunodepletion studies have suggested that this protein complex is required for proper chromosome condensation *in vitro* ^25, 26^. However, subsequent studies have challenged the view for condensin’s requirement in chromosome condensation, as chromosomes do condense to a certain degree upon condensin’s inactivation in several *in vivo* studies ^27– 32^. In addition to chromosome compaction, several studies revealed other roles for condensin in mitotic chromosome organization: maintenance of chromosomal structural integrity ^28, 30, 33^ and resolution of topological DNA entanglements ^27, 29– 31, 33^. Recent studies, using novel protein inactivation tools based on timely proteolytic cleavage of condensin complexes, revealed that condensin complexes (particularly condensin II) are indeed needed to support the structure of assembled meiotic chromosomes ^34^. It should be noted that meiotic chromosomes are very different from their mitotic counterparts, as the mono-orientation of bivalents imposes pulling forces along the entire chromosome length (rather than simply at the pericentromeric regions). Thus, it remains to be addressed if condensins are required for chromosome condensation *per se* or simply to resist mechanical stress.

Importantly, it is yet to be determined how these different functions on chromosome organization are brought about, if they result from differential activities of condensins on mitotic chromatin, or, alternatively, if a single condensin-driven reaction may account for all the reported phenotypes. *In vitro* studies revealed that condensins are able to introduce positive supercoils on circular DNAs ^35, 36^, which could account for chromosome compaction. Yet it is not clear if (and how) condensin supercoiling activity is required for *in vivo* chromosome condensation. Condensin subunits are also the major components of the chromosome scaffold ^18, 37^, and it has thus been proposed to hold chromatin loops at the central axial core of chromosomes. However, condensin I (but not condensin II) displays a highly dynamic association with mitotic chromosomes ^28, 38^, questioning the hypothesis that this complex is statically holding chromatin loops. Recent studies in budding yeast revealed that condensin complexes topologically embrace DNA molecules *in vivo* ^39^, providing strong evidence that condensins may work as an intra-chromosomal linker that brings together two distant segments of one sister chromatid and thereby promotes compaction. Further understanding on how condensin works on mitotic chromosomes is pivotal, not only to uncover the molecular mechanisms of these complexes but also to elucidate chromosome architecture itself.

### Topoisomerase II

**Figure 3.**
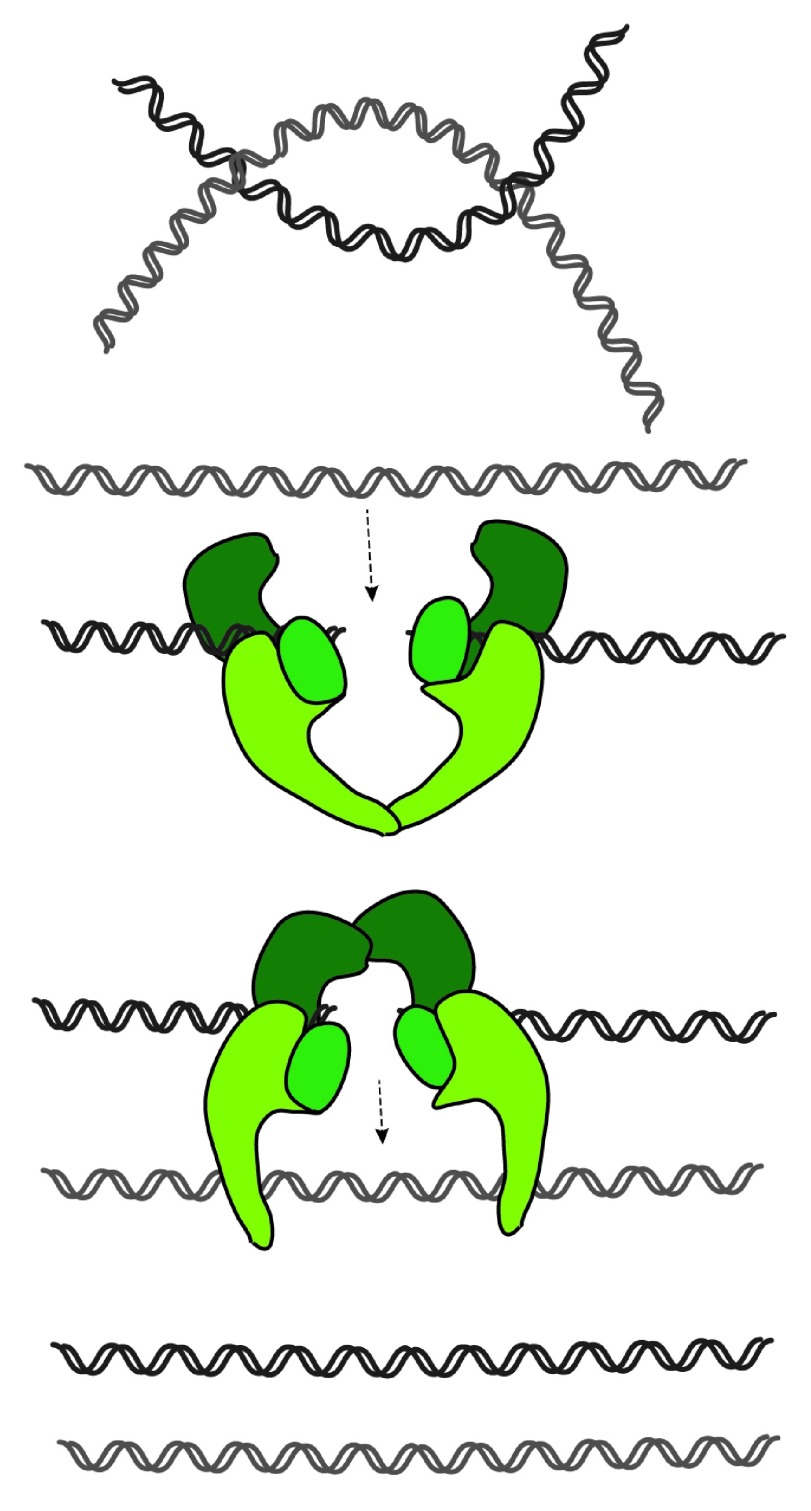
Topoisomerase II: DNA decatenation reaction driven by topoisomerase II. This enzyme cuts both strands of a DNA duplex and allows strand passage of a second duplex through the break. After strand passage, topoisomerase seals the break and releases both strands. It can thus promote the resolution of intertwines (catenations) between sister DNA molecules.

Topoisomerase II can introduce several changes in the topology of DNA molecules by driving both supercoiling and relaxing of the supercoils, and also the catenation and decatenation of DNA molecules ^40^. Although some of these reactions can be brought about by topoisomerase I, only topoisomerase II can promote the resolution of catenated sister-DNA molecules. Topoisomerase II is able to decatenate intertwined DNAs by transiently cutting both strands of a DNA molecule, which are then resealed after passage through another DNA duplex ( Figure 3). It is therefore essential for sister chromatid resolution and their efficient separation at the end of mitosis. Topoisomerase II is also a major component of the chromosome scaffold ^41^, and it has long been debatable whether or not this enzyme promotes chromosome compaction in addition (or in parallel) to sister chromatid resolution.

Topoisomerase II was reported to be dispensable for chromosome condensation in some model organisms ( *Saccharomyces cerevisiae* ^32^, *Xenopus laevis* ^42^, and human cells ^43^). Nevertheless, other studies provide evidence that topoisomerase II is necessary or at least contributes to establishing proper condensation and chromosome structure in *Schizosaccharomyces pombe* ^44, 45^, *S. cerevisiae* ^46^, *X. laevis* ^47, 48^, *Drosophila melanogaste*r ^49^, chicken ^50^, hamster ^51^, or human ^52, 53^ cells. How exactly topoisomerase II could facilitate condensation, however, remains unclear.

### Interplay between condensin I and topoisomerase II

Both condensin I and topoisomerase II localize to the central axis of mitotic chromosomes ^54, 55^ and both complexes have the ability to alter DNA topology. Thus, it has been speculated that these proteins may cooperate (directly or indirectly) in establishing chromosome compaction and organization. Condensin I was initially proposed to directly interact with topoisomerase II ^56^, but later studies failed to provide evidence for a physical interaction between these proteins ^26, 47, 57^. Nonetheless, depletion of condensins causes delocalization of topoisomerase II from the chromosome axis and decreases its decatenation activity ^54^. Recent evidence further supports the notion that during anaphase, topoisomerase is recruited to chromosome arms in a condensin-dependent manner ^58^.

Importantly, topoisomerase II was shown to be particularly efficient in decatenating (unlinking) supercoiled DNA molecules ^59^. Given the condensins’ ability to introduce positive supercoiling, it has been proposed that the topology generated by condensin I could be attracting topoisomerase II in order to drive global decatenation ^59^. This notion is further supported by studies that measure the efficiency of decatenation of circular mini-chromosomes *in vivo*, revealing that condensin promotes DNA decatenation ^57^.

In contrast to the cooperation model, other studies support the idea that condensins and topoisomerase II may have antagonistic roles in chromosome assembly. Condensins were proposed to drive lateral compaction, while topoisomerase II was suggested to induce axial compaction ^50, 60^. The question of how condensins and topoisomerase II are able to cause directional compaction within separate sister chromatids without creating new links within individual sister chromatids and tangling them together remains.

### Kif4

Kif4 is a motor protein able to bind to mitotic chromosomes. Studies in vertebrate cells reveal that Kif4 contributes to the establishment of a correct morphology and structure of chromosomes ^50, 61^. It is proposed to cooperate or work alongside condensin in shortening the lateral axis of chromosomes, possibly by creating loops of chromatin ^50^, although little is known about the molecular mechanisms in this process.

### Histone modifications

During mitosis and concomitantly with chromosome condensation, the landscape of histone modifications is altered. Histone H1, the linker histone, is hyper-phosphorylated during mitosis ^62, 63^, and it was initially thought to directly participate in condensation. However, subsequent studies suggest that histone H1 phosphorylation is not necessary for condensation ^64, 65^ but nevertheless changes the overall chromatin structure ^66, 67^. Another key mitotic histone modification is the phosphorylation of serine 10 residue of histone 3 (H3 S10) by the mitotic kinase Aurora B ^68^. The role for this modification in chromosome condensation has also been controversial ^69– 71^, although recent evidence proposes that it drives the recruitment of deacetylase Hst2, which, in turn, induces deacetylation of lysine 16 of histone 4. This change in the properties of the histone 4 tail promotes interaction with histones H2A and H2B from other nucleosomes ^72^, thereby shortening the distance between neighboring nucleosomes. This would thus support the notion that histone modifications alone can promote the condensation of chromosomes. It should be noted that several histones and histone modifications were also described to be a chromosomal “receptor” for condensin binding ^73– 76^. Thus, some histone modifications may not be a direct contributor for chromosome compaction but rather a facilitator by promoting the binding of specialized proteins that model DNA topology.

## New insights from novel approaches

### Chromosome condensation revealed by high-resolution imaging and novel quantification methods

The chromosome condensation field has been largely dominated by cytological analysis. Yet, only recently, and with the advances in imaging and imaging analysis techniques, the field has started to adopt sophisticated quantification methods to estimate changes in chromosome structure during mitosis, revealing not only the compaction state but also the kinetics of the process.

Although chromosome condensation was often thought of as a linear and gradual process, a new study suggests that in the early mitosis stages, chromosomes undergo a series of subtle compaction and expansion steps ^77^. The authors applied a series of sophisticated imaging and image analysis methods to describe changes in condensation throughout mitosis. Until mid-prophase chromosomes compact, but at late prophase stages their morphology changes and they expand at the same time sister chromatids are being individualized. This is followed by another compaction phase during prometaphase and metaphase. These observations were anticipated by a theoretical model of condensation that predicted this compaction-expansion cycle ^78^. This hypothesis assumes that compaction is causing more “stress” to chromatin, as tethering segments together induces constraints and accumulates higher potential energy. The expansion stage, therefore, releases such stress and lowers the potential energy of chromosomes. The mentioned tethers causing physical constraints could be of various natures, such as protein linkers (cohesin, condensins) or DNA catenations. The authors propose that the stress cycle is ensuring the usage of the energy stored during the early compaction events for the energy-consuming drastic changes in chromosome structure, such as individualization of sister chromatids in late prophase. A recent study, however, revealed that the resolution of sister chromatids starts early during prophase, concomitantly with chromosome compaction ^79^. The authors used sequential replication labeling with two distinct nucleotide derivatives to differentially label each DNA strand, which combined with quantitative advanced imaging allowed the assessment of the resolution process with unprecedented temporal resolution. Thus, the aforementioned compaction-expansion cycles may not necessarily correlate with differential processes throughout prophase.

In addition to the estimation of global compaction on entire chromosomes, recent quantitative microscopic assays were developed to assess local compaction ^80^. Using a fluorescent reporter to target specific loci, this study reveals that the fluorescence intensity of the reporter varies depending on the compaction stage of chromosomes – the fluorescence is 2–2.5 times higher when chromatin is less compacted (interphase) than in mitotic, condensed chromosomes. This intensity variation was caused by quenching of bound fluorophore due to changes in the local environment created by packed chromosomes. The drop in fluorescence of reporters disappears if interactions between H2A and H4 histones are abolished, suggesting that the assay is primarily sensitive to compaction at the level of neighboring nucleosomes. Therefore, it provides a convenient tool to study short-range condensation. Combining two different reporter genes along arms of a chromosome, it was possible to trace at the same time the axial (long-range) contraction of chromosomes along their longitudinal axis (distance between the reporter loci) and the short-range compaction of the marked regions. Remarkably, short-range and axial compaction have different kinetics during mitosis. In anaphase, short-range nucleosome-nucleosome compaction is happening before the axial decrease of chromosome length. Moreover, condensin depletion does not affect short-range compaction and, conversely, disturbing nucleosome-nucleosome interaction does not affect axial contraction. This led to the conclusion that short-range compaction and axial contraction are probably mostly independent and governed by different mechanisms. A common factor in both pathways is Hst2 deacetylase. By regulating H2A–H4 interaction, Hst2 promotes short-range nucleosome-nucleosome interactions and compaction. Additionally, Hst2 was shown to contribute to axial contraction by promoting condensin activity. This study proves that obtaining accurate quantification of microscopic data is very often challenging but can lead to novel discoveries.

### The minimal chromosome assembly system revealed by *in vitro* approaches

*In vitro* studies have brought major insights into many fields of biology. Separation of biological components into a controlled artificial environment with less complexity allows simpler and more precise interpretation of data. It is undeniably true that the *in vitro* results cannot be always directly translated back to the *in vivo* situation. Nevertheless, once the component or process (like chromosome condensation) is studied in the *in vitro* environment, it is easier to understand it in the *in vivo* context.

A breakthrough towards this idea was the identification of the minimal set of components that allows *in vitro* formation of a mitotic structure from uncondensed DNA in *Xenopus* egg extracts ^48^. This reductionist approach demonstrated that out of thousands of possible proteins present in metaphase extract, only six factors, when combined, are sufficient to drive effective condensation. In addition to the “usual suspects” condensins and topoisomerase II, painstaking selection of other critical components further reveals the requirement of four other factors: nucleoplasmin, Nap1, and FACT (all of them are histone chaperone proteins) and embryonic core histones. In addition, the process was shown to be ATP dependent, which is necessary for enzymatic actions of condensins and topoisomerase II. This unique approach holds the promise of providing important insights into chromosome condensation by *in vitro* perturbations.

### Lessons from studies on isolated chromosomes

Isolated entire chromosomes can be micromanipulated and subjected to measurements of their mechanical properties. This approach, pioneered using large newt chromosomes ^19, 20, 81, 82^, allows a direct measurement of the physical characteristics of chromosomes. Chromosomes can be assessed for their elastic properties in various conditions by stretching them and determining the force needed to double the chromosomal length. A major recent advance was the ability to perform similar studies on much smaller human chromosomes ^83^. Importantly, most of the prior observations were confirmed in human chromosomes, further supporting the idea that a scaffold of protein crosslinkers is not necessary to keep chromosome structure together, which is instead sustained by a network of intertwined DNA. Yet the absence of these “modulating proteins” leads to significant changes in the properties and morphology of this chromosome network.

Another *in vitro* approach has also been recently used to understand the roles of DNA catenation in human mitotic chromosomes ^84^. DNA catenations have long been speculated to be critical in mitotic chromosome structure, yet measuring DNA catenation *in vivo* has been a virtually impossible task. To test this, the authors used metaphase chromosomes isolated from human cells placed in a microfluidics lab-on-chip system, which allowed simultaneous imaging and environment control. When native metaphase chromosomes were treated with proteinase to remove all proteins, the resulting digested chromosomes were then challenged with various physical obstacles. The chromosomes preserved their canonical X-like shape and sister chromatids are kept together by thin DNA fibers in the centromeric region. Importantly, disrupting catenations, by chemical inhibition of topoisomerase II, caused drastic morphological changes along the entire length of the chromosome. Without functional topoisomerase II, the chromosomes become decondensed (elongated and rounded) and with less-defined axes along the arms. This led to the proposal that DNA catenation networks provided by topoisomerase II activity are crucial to maintain chromosome structure not only at the centromeres but also along the entire length of chromosome arms. It nevertheless remains to be determined if the same holds true *in vivo*, as it is possible that the *in vitro* manipulations may alone contribute to the observed phenotype.

### Internal chromosomal linkages revealed by Chromosome Conformation Capture methods

During interphase, chromosomes have their characteristic patterns of physical interactions of distinct regions within a single chromosome. It was recently shown in an elegant way that for the mitotic chromosome it does not really matter how it was previously folded during interphase ^85^. When cells enter mitosis, each chromosome is somehow stripped of its interphase physical contact frequency pattern and acquires a homologous physical interaction pattern throughout its entire length (no compartmentalization of interaction within itself, meaning that only short-distance interactions occur). This absence of compartmentalization in mitotic chromosomes seems to be similar in all chromosomes, regardless of the chromosome identity or the cell type. The observed interaction map was confronted with models describing the folding, dynamics, and internal organization of mitotic chromosomes. Among others, it tackles the hierarchical model of packing DNA into chromosome structure and also a long-debated existence of internal scaffold in mitotic chromosomes. The authors argue that their experimental data do not fit with hierarchical folding models while models based on the existence of 80–120 kb long loops stay with the agreement with experimental work. Unfortunately, the authors were not able to anticipate whether or not chromosome structure contains a stiff scaffold around which chromatin is organized. A model in which the folding of interphase chromatin occurs in a two-step process, nevertheless, better explains their findings. First, linear compaction occurs by creating loops of consecutive regions of the DNA of length 80–120 kb, possibly with the help of SMC complexes. The second step would be consequent axial compaction achieved by interactions of neighboring loops. It needs to be further supplemented with more detailed description of how this transition from interphase to mitotic chromosomes could be conducted inside living cells.

Biophysical modeling combined with interaction mapping has also been recently applied to study chromosome condensation in budding yeast ^86^. A computational model was built to simulate the behavior of a large DNA piece (300 kb). The chromatin was modeled as a bead-spring polymer, in which beads (nucleosome) are connected by springs (the DNA linkers between nucleosomes). Such a defined nucleosome chain was subjected to basic physics laws (Hooke’s law, Brownian movements, and others) without any additional *a priori* constraints. Simulations, further validated by *in vivo* measurements of loci proximity, indicate that yeast interphase chromatin behaves as an unconstrained nucleosome polymer. Addition of condensins (as stochastic intra-chromosomal linkers) promotes compaction of this array. Importantly, by modeling different modes for condensin binding, either connecting only two chromosomal regions or allowing interactions of two or three condensin-binding sites, the authors found that the binding of two (and no more) chromosomal regions reproduces the interaction maps found experimentally in mitotic cells. Moreover, these dynamic pair-wise interactions, in contrast to the attachment of more than two binding sites, were capable of promoting individualization of two separate DNA molecules by favoring intra-chromosomal interactions. Thus, this study further supports the notion that chromosomes may be assembled through a chromatin self-organization process, constrained by condensin interactions, rather than organized by higher order assemblies of condensin complexes within chromosomes.

## Conclusions and future perspectives

Mitotic chromosome condensation remains one of the greatest mysteries in cell biology. Recent advances in the field start to shed light onto this problem, although it is fair to assume that we are still far from understanding the rules that govern mitotic chromosome assembly. Nevertheless, recent advances to dissect metaphase chromosome compaction fail to provide solid evidence for classical models of hierarchical folding or rigid protein scaffolds at the core of chromosome assembly. A multidisciplinary perspective of the problem, combining advanced imaging with *in vivo* and *in vitro* controlled manipulations, along with biophysical studies and modeling may in the future provide an integrative view to understand how chromosomes fold at the onset of every cell division process.
